# Freeze-thaw cycles enable a prebiotically plausible and continuous pathway from nucleotide activation to nonenzymatic RNA copying

**DOI:** 10.1073/pnas.2116429119

**Published:** 2022-04-21

**Authors:** Stephanie J. Zhang, Daniel Duzdevich, Dian Ding, Jack W. Szostak

**Affiliations:** ^a^Department of Chemistry and Chemical Biology, Harvard University, Cambridge, MA 02138;; ^b^Department of Molecular Biology and Center for Computational and Integrative Biology, Massachusetts General Hospital, Boston, MA 02114;; ^c^Howard Hughes Medical Institute, Massachusetts General Hospital, Boston, MA 02114

**Keywords:** chemical nucleotide activation, RNA copying, origin of life, RNA world hypothesis, prebiotic chemistry

## Abstract

Nonenzymatic template-directed RNA copying using chemically activated nucleotides is thought to have played a key role in the emergence of genetic information on the early Earth. A longstanding question concerns the number and nature of different environments that might have been necessary to enable all of the steps from nucleotide synthesis to RNA copying. Here we explore three sequential steps from this overall pathway: nucleotide activation, synthesis of imidazolium-bridged dinucleotides, and template-directed RNA copying. We find that all three steps can take place in one reaction mixture undergoing multiple freeze-thaw cycles. Recent experiments have demonstrated a potentially prebiotic methyl isocyanide-based nucleotide activation chemistry. However, the original version of this approach is incompatible with nonenzymatic RNA copying because the high required concentration of the imidazole activating group prevents the accumulation of the essential imidazolium-bridged dinucleotide. Here we report that ice eutectic phase conditions facilitate not only the methyl isocyanide-based activation of ribonucleotide 5′-monophosphates with stoichiometric 2-aminoimidazole, but also the subsequent conversion of these activated mononucleotides into imidazolium-bridged dinucleotides. Furthermore, this one-pot approach is compatible with template-directed RNA copying in the same reaction mixture. Our results suggest that the simple and common environmental fluctuation of freeze-thaw cycles could have played an important role in prebiotic nucleotide activation and nonenzymatic RNA copying.

## Significance

The replication of RNA without the aid of evolved enzymes may have enabled the inheritance of useful molecular functions during the origin of life. Template-directed RNA copying is a posited step in RNA replication. Key steps on the path to copying of RNA templates have been studied in isolation, including chemical nucleotide activation, generation of a key reactive intermediate, and template-directed polymerization. Here we report a prebiotically plausible scenario under which these reactions can happen together under mutually compatible conditions. Thus, this pathway could potentially have operated in nature without the complicating requirement for exchange of materials between distinct environments.

Template-directed nonenzymatic RNA copying requires activated nucleotides. A range of phosphate-activating groups has been explored to chemically activate nucleotides for such polymerization reactions (1, 2). Our laboratory has demonstrated efficient copying of several short RNA templates using 2-aminoimidazole (2AI) activated ribonucleotides (2AImpN, abbreviated as *pN) (3), and shown that polymerization proceeds predominantly through spontaneously generated 5′-5′-imidazolium-bridged dinucleotides (4). Template copying is greatly enhanced in the presence of activated downstream helper oligonucleotides through the formation of monomer-bridged-oligonucleotide species that both bind more tightly to the template and are intrinsically more reactive (3, 5, 6).

Sutherland et al. (7) recently reported a potentially prebiotic route to selective phosphate activation with methyl isocyanide (MeNC), acetaldehyde, and 2AI. MeNC could have been produced in a ferrocyanide- and nitroprusside-containing environment upon ultraviolet (UV) irradiation (7). A major hurdle to the compatibility of this activation chemistry with RNA copying is that activation requires an excess of 2AI to drive the reaction forward; however, excess 2AI prevents nonenzymatic RNA polymerization by shifting the equilibrium between activated monomers and imidazolium-bridged dinucleotides in favor of activated monomers (4, 8). We have previously identified a variant of isocyanide activation chemistry, named bridge-forming activation, that circumvents this issue by directly generating bridged dinucleotides from activated mononucleotides (9). The resulting high concentration of bridged dinucleotides increases the yield of primer extension products. Although we were able to demonstrate the compatibility of bridge-forming activation with nonenzymatic RNA polymerization, bridge-forming activation depends on the presence of already activated mononucleotides. To begin to address the challenge of initial nucleotide activation under conditions that do not inhibit bridged dinucleotide accumulation, we here explore the effects of repeated freeze-thaw cycles on activation chemistry (10, 11).

Under partially frozen conditions, solutes are excluded from growing ice crystals and become concentrated in the interstitial liquid solution. This phenomenon of eutectic phase concentration has been demonstrated to facilitate the synthesis of nucleotide precursors (12), as well as the untemplated condensation of activated nucleotides into oligomers (13). Here we report the in situ MeNC-mediated activation of mononucleotides with stoichiometric 2AI by repeatedly subjecting the solution to eutectic phase freezing, which temporarily brings the reactants into close physical proximity and obviates the need for the large excess of 2AI otherwise required to drive activation in relatively dilute liquid phase reactions. We find that all four canonical ribonucleotides (as well as helper oligonucleotides) can be activated with this approach, which also yields high concentrations of bridged species. Consequently, we were also able to show that the reaction is compatible with template-directed nonenzymatic RNA copying. The geochemically plausible conditions that we have identified enable a potentially prebiotic pathway that begins with ribonucleotide 5′-monophosphates, generates 2AI activated nucleotides and then the essential bridged dinucleotides, and finally yields template-directed primer extension products, all in one reaction mixture.

## Results

**Fig. 1.**
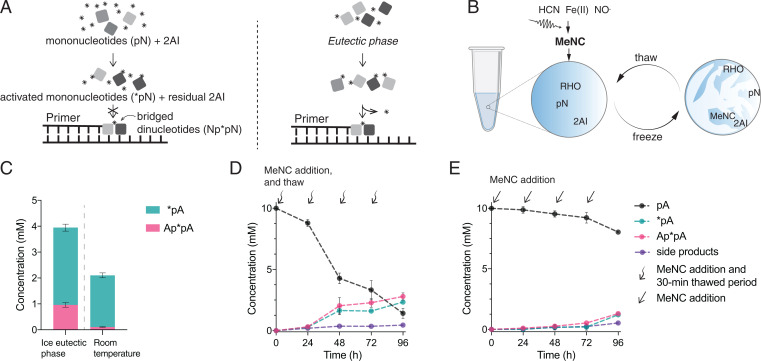
Activation in the ice eutectic phase enhances the yield of activated nucleotides (*pN) and promotes formation of bridged dinucleotides (Np*pN). (*A*) Scheme showing role of ice eutectic concentration. *Left:* At room temperature, the excess 2AI needed to drive nucleotide activation inhibits bridged dinucleotide accumulation. *Right:* activation in ice eutectic phase requires only equimolar 2AI and ribonucleotides, allowing bridged dinucleotide accumulation and template copying. (*B*) Solutes are concentrated in the ice eutectic phase. (*C*) Nucleotide activation in the ice eutectic phase (left) and at room temperature (*Right*) with one round of MeNC addition and equimolar initial nucleotides and 2AI (*SI Appendix*, Fig. S3*A*). (*D*) Cycles of MeNC addition and eutectic freezing drive continued nucleotide activation. Curvy arrows indicate addition of MeNC after each thawing of the reaction mixture. Note that the reaction mixture is frozen as eutectic ice for the entire reaction course except during the brief thawing steps (∼30 min at room temperature for the mixture to thaw completely) (*SI Appendix*, Fig. S3*B*). (*E*) Activation is inefficient at room temperature in the absence of ice eutectic freezing. Straight arrows indicate the addition of MeNC at room temperature (*SI Appendix*, Fig. S3*C*).

Addressing the incompatibility between template-directed primer extension and the initial in situ activation of mononucleotides requires a plausible pathway to nucleotide activation using a concentration of 2AI comparable to the concentration of nucleotides, rather than the large excess currently required (Fig. 1*A*). We reasoned that the ice eutectic phase as a medium for activation could temporarily increase the effective local concentration of 2AI, thereby alleviating the requirement for an absolute excess of 2AI relative to nucleotides (Fig. 1*B*). To begin exploring the effect of ice eutectic phase concentration on nucleotide activation, we treated 10 mM adenosine monophosphate (pA) with 10 mM 2AI, 100 mM 2-methylbutyraldehyde (2MBA), and 200 mM MeNC, at pH 8 under both room temperature and ice eutectic phase conditions (9). With these reactant concentrations, and incubation at room temperature, only 2.0 ± 0.1 mM activated nucleotides (∼20% of the 10 mM total available nucleotides) and only 0.11 ± 0.01 mM bridged dinucleotides were formed after 24 h (Fig. 1*C*). In contrast, using the same concentrations of reactants but subjecting the mixture to ice eutectic phase concentration at −13 °C for 24 h yielded 50% total activation, consisting of 3.0 ± 0.1mM activated nucleotides and 1.0 ± 0.1 mM bridged dinucleotides. The ice eutectic phase not only increases the yield of activated nucleotides but also acts as a storage mechanism, because activated nucleotides are less susceptible to hydrolysis at low temperatures (*SI Appendix*, Fig. S1 *C* and *D*), which further aids in the accumulation of bridged dinucleotides (Fig. 1*C*).

The possibility that in a prebiotic environment MeNC could have been released periodically in response to day-night cycles of UV irradiation (14) prompted us to test multiple cycles of eutectic phase concentration and MeNC delivery. Instead of adding 200 mM MeNC from the start, 50 mM MeNC was introduced to the solution at the beginning of each of four sequential freeze-thaw cycles. This approach further increased nucleotide activation to 75%, yielding 2.33 ± 0.01 mM activated nucleotides and 2.8 ± 0.3 mM bridged dinucleotides (Fig. 1*D*). By comparison, periodic addition of MeNC at room temperature without eutectic freezing yielded only 1.30 ± 0.6 mM activated nucleotides and 0.7 ± 0.6 mM bridged dinucleotides, or 27% total activation (Fig. 1*E*). A range of pHs was examined to rule out the possibility that freezing-induced changes in pH might account for the increase in activation (*SI Appendix*, Fig. S2).

**Fig. 2.**
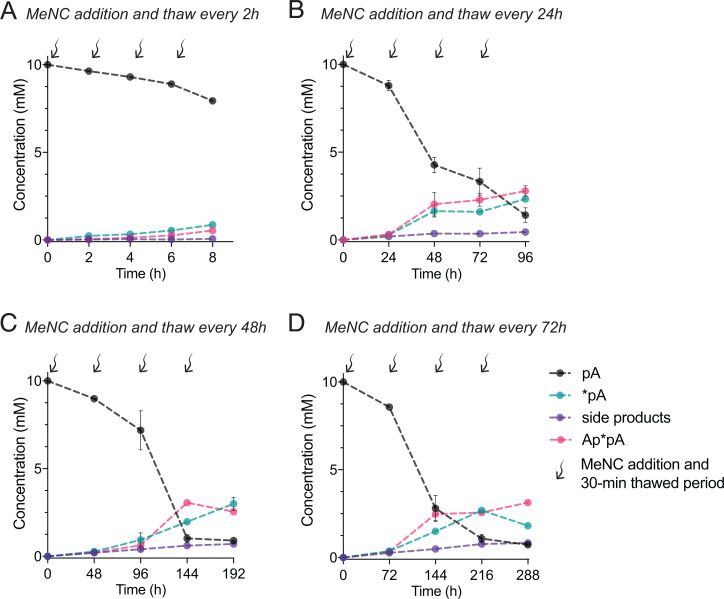
The frequency of MeNC delivery at long time intervals does not affect the overall yield of activated products. The yields of nucleotide activation were measured after each of four freeze-thaw cycles with periodic addition of MeNC. (*A*) Yields of activated species are low with time intervals on the order of hours in between MeNC additions. (*B*–*D*) However, lengthening of the time interval beyond 24 h does not significantly affect the final yield (*SI Appendix*, Fig. S5). Reaction conditions: 10 mM pA, 10 mM 2AI, 100 mM 2MBA, 50 mM MeNC, 30 mM MgCl_2_, 50 mM Na^+^-Hepes pH 8, and subsequent periodic addition of MeNC in three aliquots of 50 mM. After the last addition of MeNC, the solution was left under eutectic ice phase conditions for the indicated time, then the solution was thawed for room temperature NMR measurement.

We then varied the frequency of MeNC addition to the system and evaluated the effect on activated nucleotide yield. We found that an interval of roughly 1 day between MeNC additions led to efficient activation (Fig. 2 *A* and *B*). Further increases in the interval, up to several days, did not significantly change the activation yield (Fig. 2 and *SI Appendix*, Fig. S2 *B–D*). This not only indicates the robustness of the system in response to changes in the timing of activation but again demonstrates that freezing is a storage mechanism in our system: the accumulation of bridged dinucleotides is consistent with the expectation that activated mononucleotides and bridged dinucleotides should not readily hydrolyze in the ice eutectic state (*SI Appendix*, Fig. S1) (8, 15). Furthermore, the presence of high concentrations of bridged dinucleotides in these experiments suggests that in addition to initial nucleotide activation, bridge-forming activation is also occurring. To confirm this, we incubated 10 mM pure preactivated mononucleotides in the ice eutectic phase without additional activation chemistry and observed only 1.05 ± 0.05 mM bridged dinucleotides (*SI Appendix*, Fig. S4), compared to 2.8 ± 0.3 mM bridged dinucleotides in the presence of activation reagents. Thus, bridge-forming activation proceeds under ice eutectic phase conditions.

**Fig. 3.**
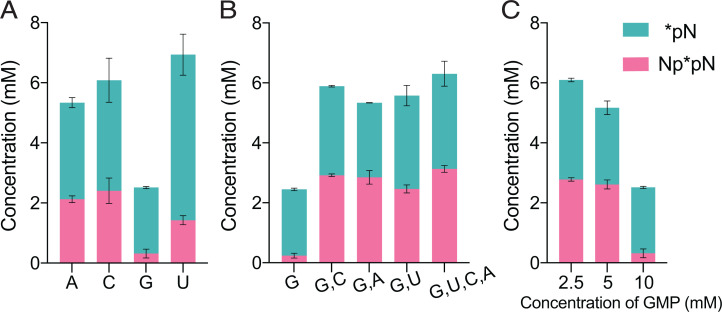
Efficient eutectic ice phase activation of all four canonical ribonucleotides. (*A*) Each of the four canonical ribonucleotides can be activated using the same approach as optimized for pA (Fig. 1*D*). However, pG exhibits lower activation compared to the other three. (*B*) All four nucleotides can be activated efficiently when incubated simultaneously. Indicated concentrations are sum totals across all the nucleotides. (*C*) GMP activation yields decrease as GMP concentration is increased, suggesting that the low activation of GMP is partly due to its low solubility in aqueous phase (compare with *A*). Total [pN] = 10 mM, 10 mM 2AI, 100 mM 2MBA, 50 mM MeNC, 30 mM MgCl_2_, 50 mM Na^+^-Hepes pH 8, and periodic addition of MeNC in three aliquots of 50 mM each (*SI Appendix*, Fig. S6).

Copying mixed sequence RNA templates requires G, C, and U in addition to A. We therefore proceeded to test the activation of all four canonical nucleotides, separately and together, under ice eutectic phase conditions. We began with 10 mM of each individual ribonucleotide and found good yields of activated species with cytidine monophosphate (pC), adenosine monophosphate (pA), and uridine monophosphate (pU). In contrast, guanosine monophosphate (pG) exhibited poor activation (Fig. 3*A*). We were able to rescue high-efficiency activation by treating a mixture of all four nucleotides (10 mM total) with activation chemistry under ice eutectic conditions (Fig. 3*B*). Dilution experiments showed that the rescue is partially due to the decreased relative concentration of pG (from 10 mM to 2.5 mM, Fig. 3*C*), suggesting that concentration-driven aggregation may interfere with the activation of pG. Our observation is another striking example of the desirability of an optimal degree of heterogeneity in prebiotic processes (16).

**Fig. 4.**
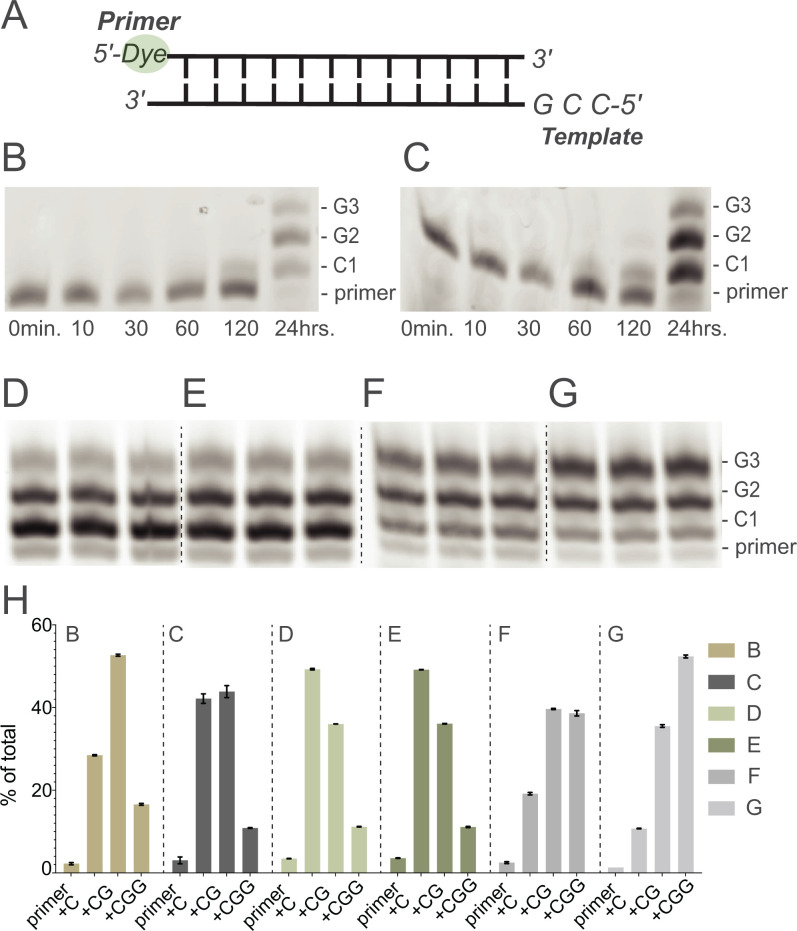
Ice eutectic phase activation enables a complete pathway from unactivated mononucleotides through activation to primer extension. (*A*) Schematic of primer-template complex. (*B*) Time course of primer extension using pure activated mononucleotides (5 mM *pG and 5 mM *pC, room temperature). (*C*) Time course of primer extension reactions using pNs (*n* = C and G) generated with MeNC activation chemistry under ice eutectic phase. (*D*) Primer-template was added to already-activated mononucleotides generated by MeNC-based nucleotide activation under ice eutectic phase conditions, yielding extended primer (as in *C*). (*E*) Primer-template duplex was directly treated with pNs (*n* = C and G) and MeNC activation chemistry under ice eutectic phase. The products of the reaction were then incubated for an additional 24 h at room temperature (*F*) without and (*G*) with bridge-forming activation. Each set of three lanes in (*D*–*G*) represents independent experimental replicates under the indicated condition. (*H*) Quantification of primer extension products. Positions of primer and +1 to +3-nucleotide extension products are indicated. Gel image of replicates for (*B*, *C*) is included in *SI Appendix*, Fig. S7.

Encouraged by these results, we sought to combine ice eutectic phase nucleotide activation and bridged species formation with template-directed primer extension (Fig. 4*A*). First, we aimed to confirm that nucleotides activated using this approach behave the same way as synthetically prepared and purified activated nucleotides. We activated 5 mM each of CMP and GMP under ice eutectic phase conditions (Fig. 3*B*) and then added the resulting mixture to a primer-template complex in solution phase. Under these conditions, the primer was extended (Fig. 4*C*), just as in the control case with purified, synthetically prepared activated mononucleotides (Fig. 4*B*). Thus, activation in the ice eutectic phase generates both activated mononucleotides and bridged dinucleotides that are functional in primer extension.

Next, we asked whether in situ activation and primer extension can occur together in a single reaction mixture during freeze-thaw cycles. In this experiment the primer-template complex was included along with all other reagents from the start and exposed to cycles of ice eutectic phase activation. We found that primer extension was comparable to that observed with pure activated mononucleotides, or with sequential eutectic activation of reactants and subsequent solution phase primer extension (compare Fig. 4*E* to *B*–*D*). We then asked whether this in situ pathway could also be combined with our previously developed bridge-forming activation. In this process isocyanide and aldehyde are added to activated mononucleotides to generate enhanced levels of bridged dinucleotides from activated mononucleotides in solution, which thereby promote primer extension. We performed in situ nucleotide activation together with primer extension as in Fig. 4*E*, but then further treated the mixture with bridge-forming activation at room temperature and incubated the mixture for an additional 24 h. As expected, primer extension was even more efficient, with 52% of the total primer extended to +3 product (Fig. 4 *G* and *H*) compared with 39% in the absence of additional bridge-forming activation (Fig. 4 *F* and *H*). This experiment demonstrates that ice eutectic phase concentration enables nucleotide activation with bridge-forming activation and primer extension, all in one reaction mixture.

**Fig. 5.**
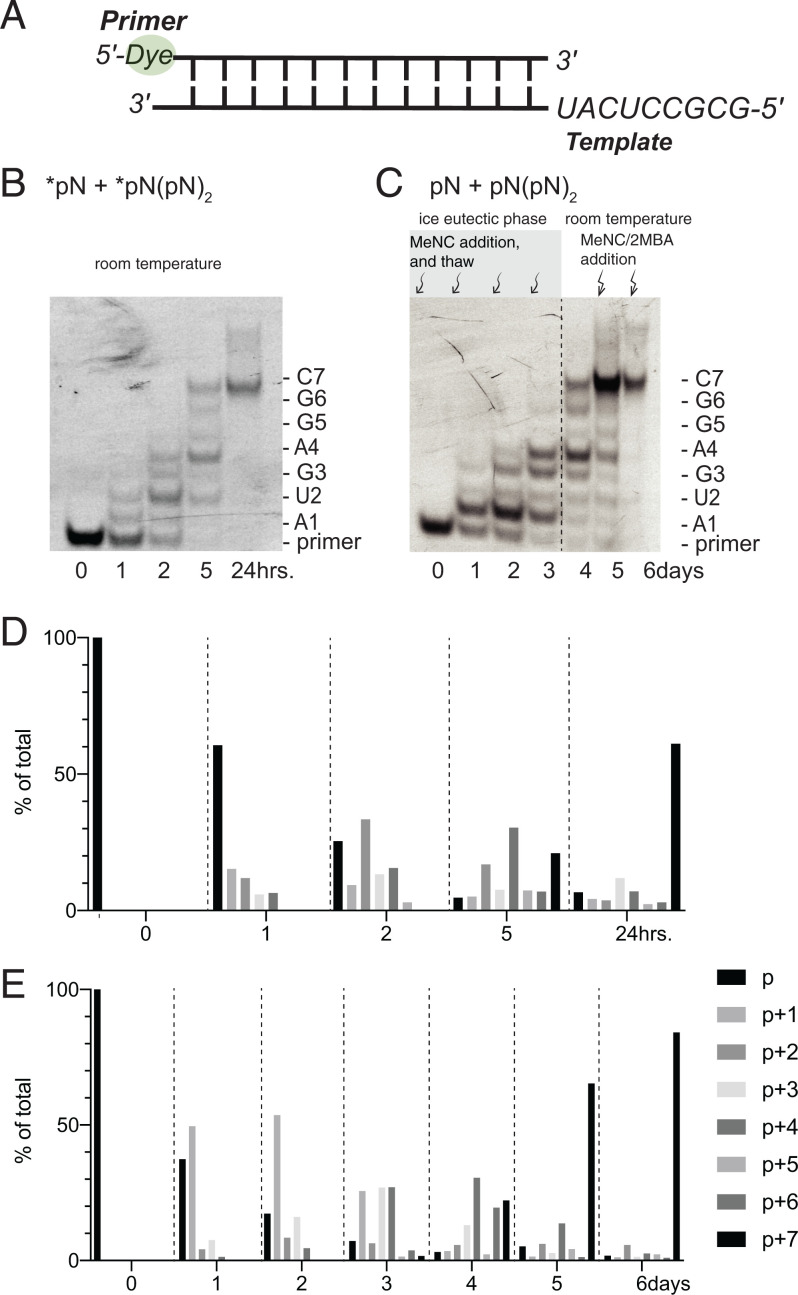
Ice eutectic phase activation enables efficient primer extension with all four nucleotides. (*A*) Schematic of primer-template complex. (*B*) Time course of primer extension using pure activated mononucleotides and trinucleotides (5 mM each *pN and 0.5 mM each *pN(pN)_2_, room temperature). (*C*) Primer-template duplex was directly treated with pNs (*n* = A, U, C, and G) and (pN)_3_ and isocyanide activation chemistry under ice eutectic phase. The products of the reaction were then incubated for 24 h at room temperature, and finally treated with additional two rounds of bridge-forming activation for another 48 h (see details in *Materials and Methods*). (*D* and *E*) Quantification of primer extension products under conditions (*B* and *C*).

The experiments discussed so far used templates with at most two different template nucleobases. To test the limits of our approach with respect to prebiotic plausibility and heterogeneity, we next asked whether the ice eutectic phase activation of all four nucleotides could be combined with bridge-forming activation and primer extension to copy mixed sequence templates. Mixed-sequence templates containing AU are known to be challenging for nonenzymatic RNA copying (17) but activated downstream helper oligonucleotides greatly facilitate primer extension on templates containing all four bases (3, 5). We have previously shown that a primer can be extended by up to seven nucleotides by copying the template sequence 3′-UACUCCGCG-5′, if six activated trimers (UGA, GAG, AGG, GGC, GCG, CGC) are included in the reaction mix (Fig. 5*A*). We repeated this experiment as a control, using 1.2 μM primer and 1.5 μM template in the presence of 5 mM of each activated mononucleotide (*pN, *n* = A, U, C, and G) and 0.5 mM of each of the six activated trimers. After 24 h, 93% of the primer had been extended by one or more nucleotides, with ∼61% of the primer extended by seven nucleotides (Fig. 5 *B* and *D*), consistent with previous results (3). In order to test monomer and helper oligonucleotide activation, the formation of imidazolium-bridged species, and primer extension all in a one pot reaction, we subjected 1.2 μM primer and 1.5 μM template, as above, along with 5 mM of each NMP and 0.5 mM of each trimer to four cycles of ice eutectic phase nucleotide activation. This procedure resulted in 97% of the primer being extended by one or more nucleotides, and 75% by four or more nucleotides, but only 22% of +7 extended product (Fig. 5 *C* and *E*). However, a subsequent 48-h incubation at room temperature with two additions of bridge-forming activation chemistry further increased the yield and extent of primer extension. After this extended incubation, 98% of the primer was extended by one or more nucleotides, 87% by four or more nucleotides, and 84% by seven or more nucleotides, an improvement over the established result with synthetically prepared reactants. Overall, this experiment demonstrates that ice eutectic phase concentration drives both monomer and trimer activation, bridged species formation, and consequently, efficient copying of RNA templates with mixed sequences containing all four canonical nucleotides. Further, the inclusion of bridge-forming activation during a subsequent liquid phase incubation consistently improves the yield of primer extension.

## Discussion

The plausibility of any scenario for the origin of life on the early Earth is greatly enhanced if different steps or stages in the overall process can occur together under mutually compatible physical and chemical conditions. There are many examples of potentially prebiotic chemical reactions that at first appeared to be incompatible but were subsequently reconciled. For example, it used to be thought that the oxygenous chemistry leading from formaldehyde to sugars such as ribose, and the nitrogenous chemistry leading from cyanide to the nucleobases were strictly incompatible due to the formation of unreactive glycolonitrile and therefore had to occur in separate environments. However, the cyanosulfidic reaction network studied by Sutherland and colleagues has shown that this is not the case, and that it is actually advantageous for these steps to occur together because the facile reduction of glycolonitrile makes it an excellent starting point for many synthetic reactions (18–20).

Resolving apparent incompatibilities between distinct aspects of prebiotic chemistry, and especially between sequential chemical reactions, is a significant challenge in origin of life studies, where the goal is to elucidate robust and realistic pathways leading from simple chemical feedstocks to replicating and evolving protocells. In the present work, we focused on one such incompatibility, namely the very high concentrations of the activating moiety 2AI thought to be required for nucleotide activation, and the fact that high concentrations of 2AI prevent the accumulation of the imidazolium-bridged species required for efficient template-directed RNA copying. We found that ice eutectic phase incubation enables nucleotide activation with equimolar concentrations of 2AI and nucleotides, thus allowing bridged dinucleotides to accumulate under a prebiotically plausible scenario. Furthermore, cycles of MeNC addition and eutectic freezing drive both efficient activation and bridged dinucleotide formation, directly enabling efficient primer extension. Thus, an environment subject to repeated cycles of freezing and thawing enables the operation of a potentially prebiotic pathway that begins with ribonucleotide 5′-monophosphates, generates 2AI activated nucleotides and then the essential bridged dinucleotides, and finally yields primer extension products, all in one mutually compatible system. Furthermore, in the presence of short helper oligonucleotides, monomer- bridged-oligonucleotide species are formed and enable the copying of mixed sequence templates (3, 5, 6). The greater affinity and higher reactivity of monomer-bridged-oligonucleotide species combine to enable the copying of templates that contain all four canonical nucleotides (6).

Our observation that the initial activation of mononucleotides can occur under ice eutectic phase conditions suggests a potential role for icy early Earth environments in nonenzymatic RNA copying. The formation and thawing of ice is a common phenomenon on Earth’s surface and can take place during day-night temperature cycles, seasonal temperature changes, or random weather-based changes (21, 22). Ice has previously been implicated in other relevant phenomena, including promoting processes from prebiotic synthesis (12) to RNA copying, and may even have astrobiological implications (23, 24). We observed that the efficiency of nucleotide activation is robust with respect to changes in the length of freeze-thaw cycles and the frequency of MeNC addition. MeNC is thought to have been released from prebiotic feedstocks exposed to UV irradiation from the Sun (7). The relative independence of activation efficiency from the frequency of MeNC delivery suggests that the required temperature cycling conditions are not stringently constrained, and therefore any corresponding early Earth environment need not be highly contrived. Ice eutectic-based nucleotide activation may not even have been required continuously: once the initial pool of activated mononucleotides had been generated, the application of solution-phase bridge-forming activation could slowly reactivate hydrolyzed nucleotides as well as regenerate bridged dinucleotides in solution. Additional experiments will be needed to determine how long this system can replenish hydrolyzed activated nucleotides without the need for additional freezing after the initial activation. It is possible that although the ice eutectic phase concentration favors activation chemistry, the low temperature may inhibit the actual primer extension reaction. Indeed, primer extension during the freeze-thaw phase of our experiments may be occurring primarily during the relatively short but nonnegligible thaw intervals. Nucleotide activation during cycles of partial drying and rehydration may also be relevant (25).

The key ingredient in the chemical nucleotide activation procedure that we used is MeNC, so it is important to consider whether MeNC could be generated in an environment compatible with our multistep pathway. For example, the strong UV absorption of nucleotides might leave insufficient UV light for MeNC generation. However, nucleotides absorb UV light most strongly between 240 nm and 280 nm, whereas the generation of MeNC proceeds with longer wavelength irradiation (26). Specifically, two steps in the plausible synthesis of MeNC exploit the use of UV light: the production of nitroprusside has been achieved with 365 nm or 254 nm light and isocyanide ligand exchange also occurs during exposure to 365 nm light (7), consistent with the broad band irradiation from the young Sun (27). The formation of MeNC using longer wavelength UV irradiation suggests that the generation of MeNC is compatible with the presence of nucleotides. Another consideration is that an important ingredient in MeNC synthesis is methylamine, which others have suggested could have been delivered to the primordial Earth by comets or formed by reduction of HCN with hypophosphite and sponge nickel (7, 28). On the basis of Ugi-type reactions (29), a primary amine such as methylamine will readily react with an aldehyde, forming an iminium ion. However, this may not affect the nucleotide activation chemistry because MeNC will still add reversibly to the iminium ion to produce the nitrilium ion required for nucleotide activation (14). In addition, 2-hydroxy-*N*-methylpropanamide, the side product of nucleotide activation, will eventually hydrolyze to regenerate methylamine, which can then take part in another round of MeNC synthesis (7). The prebiotic plausibility of the remaining chemical ingredients has been previously discussed (18, 20, 30), but additional experiments will be needed to reconstitute in situ MeNC generation with simultaneous nucleotide activation.

## Materials and Methods

### General Information.

#### Materials.

Reagents and solvents were obtained at the highest available purity from Acros Organics, Alfa Aesar, Fisher Scientific, Sigma-Aldrich, ThermoFisher Scientific, or Tokyo Chemical Industry Co., and were used without further purification unless noted below. Nucleoside-5′-monophosphates, free acid, were purchased from Santa Cruz Biotechnology. 2-aminoimidazole hydrochloride and 2,2′-dipyridyldisulfide were purchased from Combi Blocks. The 5′-phosphorylated trimers, 5′-aminoimidazolium activated trimers, and the 5′-aminoimidazolium activated mononucleotides were prepared as previously described (3). RNA oligonucleotides for standard primer extension experiments were purchased from Integrated DNA Technologies. The synthesis and storage of methyl isocyanide was as previously described (9). All reactions were carried out in DNase/RNase-free distilled water.

#### NMR spectroscopy.

^1^H- and ^31^P-NMR spectra were obtained using a Varian INOVA NMR spectrometer operating at 400 MHz and 161 MHz, respectively. Samples in H_2_O/D_2_O mixtures were analyzed using Wet1D suppression to collect ^1^H-NMR data. Chemical shifts (δ) are shown in parts per million. Coupling constants (J) are given in hertz (Hz) and the notations s, d, t, and m represent singlet, doublet, triplet, and multiplet multiplicities, respectively.

#### pH measurements.

pH values were determined by a micro pH probe (Orion 9863BN) equipped with a needle tip and a SevenCompact meter (Mettler Toledo S220).

#### Data analysis.

All NMR spectra were analyzed using MestReNova (version 12.0.3). The yields of conversion were determined by the relative integration of the signals in the ^1^H or ^31^P-NMR spectra.

### Preparation, Storage, and Concentration Determination of Stock Solutions.

Stock solution of nucleoside-5′-monophosphate disodium salt was prepared in DNase and RNase-free distilled water. After adjusting the pH to the reported values with NaOH/HCl, the stock solution was filtered with 0.22-μm syringe filters (Millipore Sigma). Each stock solution was then aliquoted and kept at −20 °C until further use. Stock solution of 2-aminoimidazole hydrochloride was prepared in DNase and RNase-free distilled water, filtered with a 0.22-μm syringe filter (Millipore Sigma), and kept at −20 °C until further use. The pH was adjusted to the reported values with NaOH/HCl right before the reaction to prevent possible polymerization at basic pH.

The concentrations of the nucleoside-5′-monophosphate solutions were determined by analysis of serial dilutions on a UV spectrophotometer. The absolute concentrations of other stock solutions were determined by comparing the integrals of ^1^H-NMR peaks of interest to the calibrant, trimethyl phosphate, by NMR spectroscopy.

### Sample Preparation for Ice Eutectic Phase Experiments.

Reaction mixtures were first flash-frozen by immersion in liquid nitrogen, followed by incubation at −13 °C in a standard laboratory freezer unit for the indicated time (11, 15, 31).

In Fig. 1*,* reaction conditions are as follows: 10 mM pA, 10 mM 2AI, 100 mM 2MBA, 30 mM MgCl_2_, 50 mM Na^+^-Hepes pH 8, plus (Fig. 1*C* ) one addition of 200 mM MeNC at ice eutectic phase or room temperature, or (Fig. 1*D*) periodic addition of the MeNC beyond the initial 50 mM in three aliquots of 50 mM each under ice eutectic phase at −13 °C or (Fig. 1*E*) at room temperature.

### Primer Extension Reactions Using Only Monomers.

#### Control primer extension reactions.

The primer-template duplex was first annealed in a 40 μL solution containing 2 μM thiol-modified primer (/5ThioMC6-D/AGUGAGUAACUC, IDT; see *Sample preparation and food analysis* for use of the thiol to dye-label products), 3.1 μM template (CCGGAGUUACUCACU, IDT), 125 mM Na^+^-Hepes (pH 8.0), and 62.5 mM MgCl_2_ by heating at 95 °C for 3 min followed by cooling to 23 °C at a rate of 0.1 °C/s. The reaction was initiated by the addition of 10 μL 100 mM activated mononucleotides.

#### Eutectic phase primer extension reactions.

The primer-template duplex was first annealed in a 100 μL solution containing 2.4 μM thiol-modified primer (/5ThioMC6-D/AGUGAGUAACUC, IDT), 3.0 μM template (CCGGAGUUACUCACU, IDT), 125 mM Na^+^-Hepes (pH 8.0) by heating at 95 °C for 3 min followed by cooling to 23°C at a rate of 0.1 °C/s. The reaction was initiated by the addition of 100 μL mixture of 10 mM pN, 10 mM 2AI, 100 mM 2MBA, 30 mM MgCl_2_, 50 mM Na^+^-Hepes pH 8, after four cycles of ice eutectic phase activation (50 mM MeNC each addition). For bridge-forming activation, the reaction mixture was incubated at room temperature with an additional 100 mM 2MBA and 200mM MeNC for 24 h.

### Trimer-Assisted Primer Extension Reactions.

#### Control primer extension reactions.

The primer-template duplex was first annealed in a 40 μL solution containing 3.6 μM thiol-modified primer (/5ThioMC6-D/CGCUCGACUG, IDT), 4.5 μM template (5′ GCGCCUCAUCAGUCGAGCG, IDT), 125 mM Na^+^-Hepes (pH 8.0), and 62.5 mM MgCl_2_ by heating at 95 °C for 3 min followed by cooling to 23 °C at a rate of 0.1 °C/s. The final reaction mixture contained 0.5 mM each activated trimer and 5 mM each activated monomer.

#### Eutectic phase primer extension reactions.

The primer-template duplex was first annealed in a 100 μL solution containing 2.4 μM thiol-modified primer (/5ThioMC6-D/AGUGAGUAACUC, IDT), 3.0 μM template (CCGGAGUUACUCACU, IDT), 125 mM Na^+^-Hepes (pH 8.0) by heating at 95 °C for 3 min followed by cooling to 23°C at a rate of 0.1 °C/s. The reaction was initiated by the addition of 100 μL mixture of 5 mM each pN, 0.5 mM each (pN)_3_, 23 mM 2AI, 100 mM 2MBA, 50 mM MgCl_2_, 50 mM Na^+^-Hepes pH 8, and periodic addition of MeNC in four aliquots of 50 mM each under ice eutectic phase. The reaction mixture was then further incubated with an additional 100 mM 2MBA and 100 mM MeNC on day 5 and day 6.

### Sample Preparation and Product Analysis.

#### Sample purification and preparation.

Ten-microliter aliquots were removed at given time points and desalted using a ZYMO Oligo Clean & Concentrator spin column (ZYMO Research) (adapted from ref. 9). The isolated material was resuspended in 30 μL 100 mM Na^+^-Hepes (pH 7.50) and disulfide bonds were reduced using a 10-fold molar excess of Tris-(2-carboxyethyl) phosphine hydrochloride. Alexa 488 C5 maleimide dissolved in anhydrous dimethyl sulfoxide at a concentration of 1 mM was added to the primer-template duplex dropwise, and the coupling reaction was allowed to proceed in the dark at room temperature for 2 h. The labeled primer-template duplex was separated from free dye using ZYMO DNA Clean & Concentrator spin columns, resuspended in 5 μL of 100 mM Na^+^-Hepes buffer, and mixed with 30 μL of quenching buffer containing 8.3 M urea, 1.3 × Tris-borate-EDTA buffer (pH 8.0), 0.005% bromophenol blue, 0.04% Orange G, and 75 μM RNA complementary to the template. To denature the labeled product from the template prior to polyacrylamide gel analysis, and sequester the template with its complement (added in excess as a competitor), the sample was heated to 95 °C for 3 min. and cooled to 23 °C at 0.1 °C/s.

#### Analysis of the polymerization products.

Twenty percent polyacrylamide gels were prepared using the SequaGel–UreaGel system (National Diagnostics, Atlanta, GA). The gels were allowed to prerun at a constant power of 20 W for 40−45 mins. Fifteen-microliter aliquots of samples were separated by 20% (19:1) denaturing polyacrylamide gel electrophoresis at a constant power of 5 W for 10 min and then 25 W for 1.5 h. Gels were imaged on a Typhoon 9410 scanner (GE Healthcare, Little Chalfont, Buckinghamshire, UK) and quantified using the accompanying ImageQuant TL software. All reactions were performed in duplicate or greater as indicated.

## Supplementary Material

Supplementary File

## Data Availability

Raw gel and NMR data are available at the Open Science Framework (OSF): https://osf.io/sjtwu/?view_only=c38e3a8bced14c2880fb5323d9f12891.
